# The association between soluble intercellular adhesion molecule-1 levels in drained dialysate and peritoneal injury in peritoneal dialysis

**DOI:** 10.1080/0886022X.2017.1287735

**Published:** 2017-02-15

**Authors:** Yusuke Igarashi, Yoshiyuki Morishita, Hiromichi Yoshizawa, Reika Imai, Toshimi Imai, Ichiro Hirahara, Tetsu Akimoto, Susumu Ookawara, Kenichi Ishibashi, Shigeaki Muto, Daisuke Nagata

**Affiliations:** a Division of Nephrology, Department of Internal Medicine, Jichi Medical University, Shimotsuke City, Tochigi, Japan;; b Division of Nephrology, Department of Integrated Medicine, Saitama Medical Center, Jichi Medical University, Omiya, Saitama City, Saitama, Japan;; c Department of Medical Physiology, Meiji Pharmaceutical University, Kiyose, Tokyo, Japan

**Keywords:** Drained dialysate, ICAM-1, peritoneal dialysis, peritoneal injury, sICAM-1

## Abstract

**Background:** Chronic inflammation of the peritoneum causes peritoneal injury in patients on peritoneal dialysis. Intercellular adhesion molecule-1 and its circulating form, soluble intercellular adhesion molecule-1, play pivotal roles in inflammation. However, their role in peritoneal injury is unclear.

**Methods:** We measured changes in intercellular adhesion molecule-1 expression in the peritoneum of a peritoneal injury model in rats. The associations between soluble intercellular adhesion molecule-1 levels in drained dialysate and the solute transport rate (D/P-Cr and D/D0-glucose) determined by the peritoneal equilibration test, and matrix metalloproteinase-2 levels in drained dialysate were investigated in 94 peritoneal drained dialysate samples.

**Results:** Intercellular adhesion molecule-1 expression was increased in the peritoneum of rats with peritoneal injury. Soluble intercellular adhesion molecule-1 levels in drained dialysate were significantly positively correlated with D/P-Cr (*r* = .51, *p* < .01) and inversely correlated with D/D0-glucose (*r* = −.44, *p* < .01). They were also significantly positively correlated with matrix metalloproteinase-2 levels in drained dialysate (*r* = .86, *p* < .01).

**Conclusions:** Intercellular adhesion molecule-1expression is increased in the peritoneum of a peritoneal injury model in the rat, and soluble intercellular adhesion molecule-1 levels in drained dialysate are associated with peritoneal injury in patients on peritoneal dialysis. These results suggest that soluble intercellular adhesion molecule-1 could be a novel biomarker of peritoneal injury in patients on peritoneal dialysis.

## Introduction

Peritoneal dialysis (PD) is one of the home-based renal replacement therapies for end-stage renal disease.^1^ PD has the advantage of maintaining residual renal function. PD results in a lower mortality rate in younger patients without comorbidities for several years after induction of renal replacement therapy compared with hemodialysis.^2^ However, a critical problem of PD is that it cannot be continued in the long term (usually less than 10 years) owing to peritoneal injury.^2^ Peritoneal injury is characterized by an increased thickness of the peritoneal membrane, predominantly in the submesothelial collagenous area, decreased ultrafiltration, and an increased solute transport rate.^3^ Peritoneal injury leads to withdrawal from PD and may occasionally cause encapsulating peritoneal sclerosis, which is a severe, life-threatening complication in PD.^4^ Therefore, periodical monitoring of peritoneal injury is important in PD. The solute transport rate is measured as an estimator of peritoneal function and peritoneal injury by the peritoneal equilibration test (PET).^5^ However, the PET is an invasive and time-consuming method because it requires repeated blood and peritoneal drained dialysate sampling during 4 h.^5^ To improve these problems, several molecules, including interleukin-6, cancer antigen 125, and matrix metalloproteinase-2 (MMP-2), have been studied as biomarkers for peritoneal injury. However, these potential biomarkers are still under investigation for clinical use.^6^^,^^7^ Therefore, further investigation and identification of biomarkers for peritoneal injury are important for improving the prognosis of patients on PD.

Chronic inflammation of the peritoneum is a major contributor for developing peritoneal injury in PD.^8^ Many factors, including uremic condition, PD solution, PD catheters, and acute infectious peritonitis, have been reported to cause chronic inflammation of the peritoneum, which leads to peritoneal injury in patients on PD.^9^

Intercellular adhesion molecule-1 (ICAM-1), a glycoprotein, is expressed in various cells, including epithelial cells, mesothelial cells, endothelial cells, lymphocytes, monocytes, and fibroblasts.^10^ ICAM-1 has a pivotal role in most inflammatory reactions by adhering inflammatory cells to inflammatory sites.^11^ Soluble ICAM-1 (sICAM-1) is a circulating form of ICAM-1.^12^ sICAM-1 is increased in inflammation and found in various body fluids, such as serum, sputum, synovial fluids, and urine.^12^ Therefore, sICAM-1 is a potential biomarker for various diseases, such as cardiovascular disease, cancer, neurological disorders, and transplantation and graft rejection in which chronic inflammation has important roles.^12^ However, the association between sICAM-1 expression levels in drained dialysate and peritoneal injury is unknown.

Based on the above-mentioned findings, we hypothesized that ICAM-1 expression of the peritoneum is increased in peritoneal injury, and its circulating form, sICAM-1, in drained dialysate may be a useful biomarker of peritoneal injury in patients on PD. To test this hypothesis, we investigated ICAM-l levels in the peritoneum using a rat peritoneal injury model. We also investigated the associations between sICAM-1 levels in drained dialysate and the solute transport rate as estimated by the PET, MMP-2 levels in drained dialysate, and other parameters in patients on PD.

## Methods

This study was performed in accordance with the Declaration of Helsinki and was approved by the ethics committee of Jichi Medical University. Written informed consent was obtained from all enrolled patients. This study was registered with the University Hospital Medical Information Network Clinical Trial Registry (UMIN-CTR; identification number: UMIN000014124). Animal experimental protocols were approved by the animal ethics committee of Jichi Medical University and were conducted in accordance with the Use and Care of Experimental Animals Guidelines from the Jichi Medical University Guide for Laboratory Animals.

### Rat model of peritoneal injury

We used the methylglyoxal-induced model of peritoneal injury using rats. The details have been described previously.^13^ Briefly, Sprague–Dawley male rats, aged 12 weeks and weighing 230–250 g, were intraperitoneally injected with 100 ml/kg PD solution containing 20 mM methylglyoxal (Sigma-Aldrich, St. Louis, MO) using an injection syringe. This single injection was repeated 5 d a week for 3 weeks. The PD solution contained 2.5% glucose, 100 mM NaCl, 35 mM sodium lactate, 2 mM CaCl_2_, and 0.7 mM MgCl_2_ (Midperic, Terumo, Tokyo, Japan). The following groups served as controls: rats without any treatment (mock rats) and rats injected with peritoneal dialysis fluid without methylglyoxal (control rats).

### Quantitative real-time reverse-transcription polymerase chain reaction (qRT-PCR)

Details of qRT-PCR have been described previously.^14^ Briefly, peritoneal sections that were purified from rats were homogenized using a glass homogenizer and a filter column shredder (QIA shredder; Qiagen, Valencia, CA). RNA from the peritoneum was then isolated using the RNeasy kit (Qiagen, Valencia, CA). A total of 1 μg of isolated RNA was reverse-transcribed using the Superscript III first-strand synthesis system (Thermo Fisher Scientific, Waltham, MA). qRT-PCR was performed using the SYBR Green ER qPCR super mix (Thermo Fisher Scientific, Waltham, MA). ICAM-1 mRNA expression levels were normalized against glyceraldehyde-3-phosphate dehydrogenase (GAPDH) as the endogenous control. Primers for rat GAPDH and ICAM-1 were purchased from Takara Bio (Otsu, Shiga, Japan). The sequences of ICAM-1 were 5′-GCTTCTGCCACCATCACTGTGTA-3′ (sense strand) and 5′-ATGAGGTTCTTGCCCACCTG-3′ (antisense strand), and those of GAPDH were 5′-GGCACAGTCAAGGCTGAGAATG-3′ (sense strand) and 5′-ATGGTGGTGAAGACGCCAGTA-3′ (antisense strand). Data are expressed as relative quantities compared with mock rats.

### Histological analysis

Histological analysis of the rodents peritoneal injury model has been described previously.^14^ Briefly, peritoneal sections were subjected to Azan staining to evaluate the intensity of fibrotic changes. For evaluation of the degree of peritoneal thickening, the thickness of the submesothelial compact zone was measured in 10 fields of each Azan-stained sample, which was chosen randomly at a magnification of ×200, using computerized image analysis software (Image Pro 5.1; Media Cybernetics, Rockville, MD). The thickness of the submesothelial compact zone was identified as the membrane area extending from the lower limit of the mesothelial layer to the upper limit of the muscle layer. The average thickness of the submesothelial compact zone in each rat was calculated and defined as the peritoneal thickness.

### Immunohistochemistry

A Histofine kit (Nichirei Biosciences Inc., Tokyo, Japan) was used for immunohistochemistry. The details of immunohistochemistry analysis have been described previously.^15^ Briefly, paraffin-embedded sections of the peritoneum were deparaffinised, rehydrated, and autoclaved to retrieve antigens. Endogenous peroxidase activity was blocked by 3% H_2_O_2_ in methanol for 10 min at room temperature. Sections were incubated overnight at 4 °C with 1:100 dilution of anti-ICAM-1 monoclonal antibody (Santa Cruz Biotechnology, Dallas, TX) in phosphate-buffered saline. After sections were rinsed in phosphate-buffered saline, they were incubated with horseradish peroxidase-coupled goat antibody. A diaminobenzidine reaction was performed using a liquid diaminobenzidine substrate-chromogen system (Nichirei Biosciences Inc., Tokyo, Japan). Immunolabeled sections were examined with a light microscope and processed with Adobe Photoshop software (Adobe Systems, San Jose, CA).

### Patients on PD and drained dialysate samples

From June 2006 to February 2014, 94 drained dialysate samples from 50 patients on PD (37 men and 13 women who were aged 57.0 ± 14.1 years) were analyzed. The duration of PD was 1.9 ± 1.4 years. The number of patients on PD who had a history of acute infectious peritonitis was 14 (28%). The number of patients on PD who had diabetes mellitus was 13 (26%). The other characteristics of enrolled patients on PD are shown in Table 1.

**Table 1.** Patients' baseline characteristics (*n* = 50).

Age (years)	57.0 ± 14.1		
Sex			
Male	37		
Female	13		
Body mass index (kg/m^2^)	23.6 ± 4.0		
Duration of peritoneal dialysis (years)	1.9 ± 1.4		
History of peritonitis			
(+)	14		
(−)	36		
Diabetes mellitus			
(+)	13		
(−)	37		
Initial nephropathy			
Chronic glomerulonephritsis	20		
Diabetic nephropathy	12		
Renal screlosis	8		
Lupus nephritis	1		
Alport syndrome	1		
Unknown	8		
Systolic blood pressure (mmHg)	147.5 ± 21.9		
Diastolic blood pressure (mmHg)	86.5 ± 13.2		
Hemoglobin (g/dl)	10.8 ± 1.2		
Albumin (g/dl)	3.4 ± 0.5		
CRP (g/dl)	0.2 ± 0.3		

CRP: C-reactive protein.

### The PET for patients on PD

For performing the PET, we obtained samples from drained dialysate and blood from PD patients. All the samples were obtained from patients on PD who had been free of signs of peritonitis for at least 1 year. Details of the PET have been described previously.^16^ Briefly, intra-abdominal PD solution was drained, and PD solution containing 2.5% glucose was injected intraperitoneally. The creatinine (Cr) level in the drained dialysate, which was obtained 4 h after injection (D), was divided by that in plasma (P) to obtain the D/P-Cr ratio. The glucose level in the drained dialysate, which was obtained 4 h after injection (D), was divided by that obtained immediately after injection of PD solution (D0) to obtain D/D0 glucose.

### Enzyme-linked immunosorbent assay for sICAM-1 and MMP-2

The concentrations of sICAM-1 in drained dialysate that were obtained at the PET were measured by enzyme-linked immunosorbent assay (ELISA) using the human sICAM-1 ELSIA kit (RayBiotech, Inc., Norcross, GA) according to the protocol of the manufacturer. A volume of 100 μl of drained dialysate was added to each plate, which was pre-coated with an anti-human sICAM-1 antibody. The plates were incubated for 2.5 h at room temperature with gentle shaking. After washing four times with 300 μl wash solution, 100 μl biotinylated anti-human sICAM-1 antibody solution was added to each well and incubated for 1 h at room temperature with gentle shaking. After washing four times with 300 μl wash solution, 100 μl horseradish peroxidase-conjugated streptavidin solution was added to each well and incubated for 45 min at room temperature with gentle shaking. After washing four times with 300 μl wash solution, 100 μl 3,3′,5,5′-tetramethylbenzidine substrate was added to each well, and incubated for 30 min at room temperature with gentle shaking. A volume of 50 μl stop solution was then added to each well and the resultant color was read at 450 nm.

MMP-2 concentrations in drained dialysate that was obtained at the PET were also measured by ELISA. MMP-2 ELISA was conducted using the MMP-2 ELISA kit (GE Healthcare, Piscataway, NJ) according to the protocol of the manufacturer. Briefly, bound MMP-2 was detected using a peroxidase-labeled Fab’ antibody to MMP-2 and any excess was removed by washing and aspiration. The amount of peroxidase bound to each well was determined by addition of 3,3′,5,5′-tetramethylbenzidine substrate. The reaction was stopped and the resultant color was read at 450 nm.

### Laboratory methods

The other blood parameters were determined by the Department of Clinical Laboratory, Jichi Medical University.

## Statistical analysis

All data are expressed as the mean ± standard error (SE). The means of two different groups were compared using the *t*-test. ANOVA was used to investigate differences among groups. If statistical significance was detected by ANOVA, Tukey’s test was performed as a *post hoc* analysis to compare the means of two different groups. Relationships between continuous variables were analyzed using Pearson’s correlation tests or linear regression analysis. Differences with a *p* values < .05 were considered significant.

## Results

### Histological changes in the peritoneum in rats with peritoneal injury

**Figure 1.**
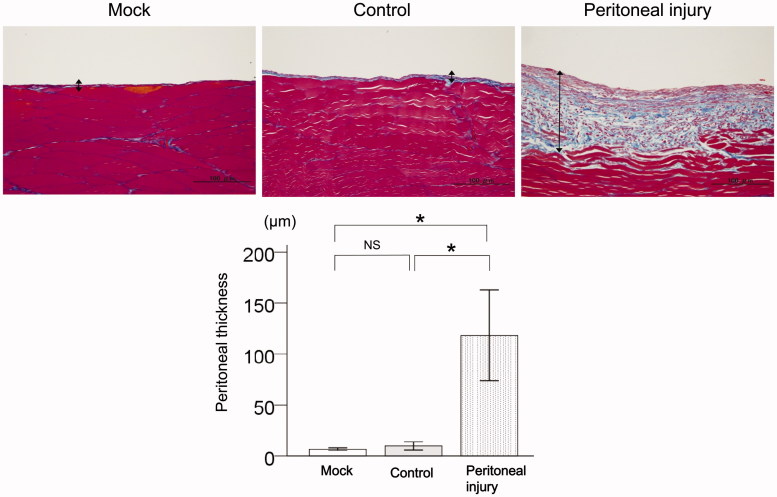
Histological changes in the peritoneum of rats with peritoneal injury. Representative Azan staining of the peritoneum with fibrous thickness indicated by arrows. Quantitative analysis of peritoneal thickness in mock rats (*n* = 6), control rats (*n* = 6), and rats with peritoneal injury (*n* = 6). Values are the mean ± standard error (error bars). Scale bar =100 μm. NS: not significant. *p < .05.

Significant peritoneal fibrous thickening was observed in rats with peritoneal injury compared with mock rats (*p* < .01) and control rats (*p* < .01, Figure 1). There was no significant difference in peritoneal fibrous thickness between mock rats and control rats (Figure 1).

### ICAM-1 expression in the peritoneum of rats with peritoneal injury

**Figure 2.**
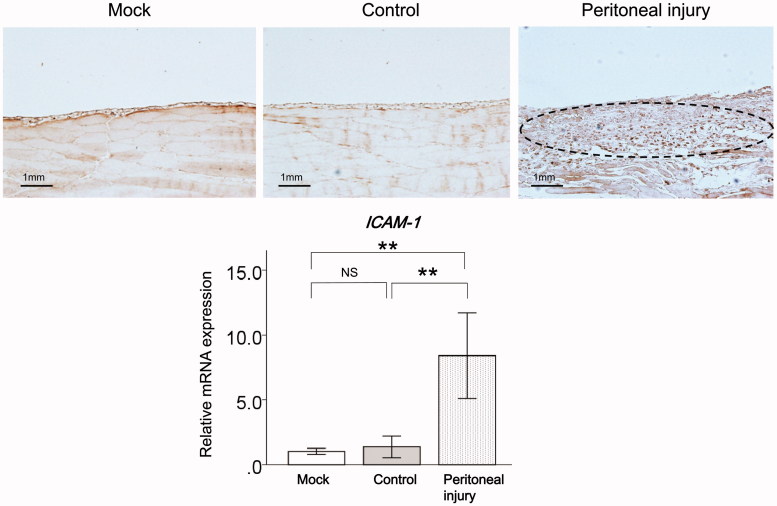
ICAM-1 expression in the peritoneum of rats with peritoneal injury. (A) Representative immunostaining for ICAM-1 (broken lines) in the peritoneum of mock rats (*n* = 6), control rats (*n* = 6), and rats with peritoneal injury (*n* = 6). (B) qRT-PCR analysis of ICAM-1 mRNA expression in the peritoneum of mock rats (*n* = 6), control rats (*n* = 6), and rats with peritoneal injury (*n* = 6). Values are mean ± standard error (error bars). ICAM-1: intercellular adhesion molecule-1; qRT-PCR: quantitative real-time reverse-transcription polymerase chain reaction; NS: not significant. **p < .01.

ICAM-1 mRNA expression levels were significantly higher in the peritoneum of injured rats compared with mock rats (*p* < .01) and control rats (*p* < .01, Figure 2). There was no significant difference in ICAM-1 expression levels in the peritoneum between mock rats and control rats (Figure 2).

### Correlations between sICAM-1 levels in drained dialysate and results of the PET in patients on PD

**Figure 3.**
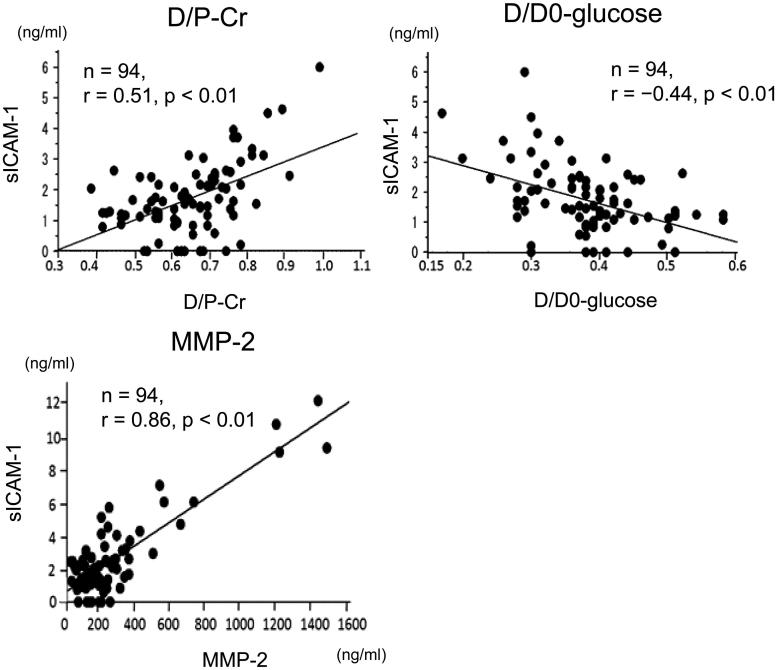
Correlations between sICAM-1 levels in drained dialysate and results of the PET and MMP-2 levels in drained dialysate in patients on PD. Correlations between sICAM-1 levels in drained dialysate and D/P-Cr, D/D0-glucose, which was estimated by the PET, and MMP-2 levels in drained dialysate in patients on PD. sICAM-1: soluble intercellular adhesion molecule-1; PET: peritoneal equilibration test; MMP-2: matrix metalloproteinase-2; PD: peritoneal dialysis; D/P-Cr: dialysate-to-plasma ratio of creatinine; D/D0-glucose: the ratio of dialysate glucose concentrations at 4 and 0 h.

Levels of sICAM-1 in drained dialysate were significantly positively correlated with D/P-Cr (*n* = 94, *r* = .51, *p* < .01) (Figure 3) and inversely correlated with D/D0-glucose (*n* = 94, *r* = −.44, *p* < .01) in patients on PD (Figure 3). Levels of sICAM-1 in drained dialysate were significantly positively correlated with MMP-2 levels in drained dialysate (*n* = 94, *r* = .86, *p* < .01) in patients on PD (Figure 3).

### Correlations between sICAM-1 levels in drained dialysate and parameters of patients on PD

**Figure 4.**
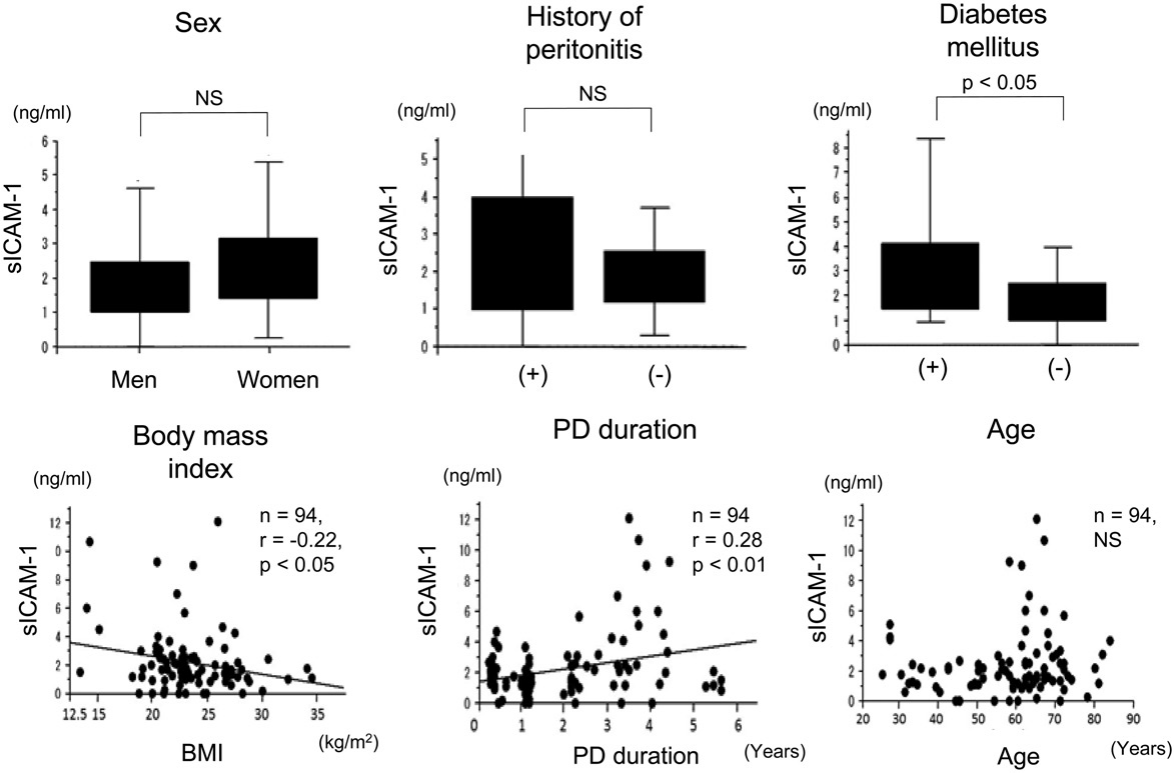
Correlations between sICAM-1 levels in drained dialysate and other parameters of patients on PD. Differences in sICAM-1 levels in drained dialysate according to sex, episodes of acute inflammatory peritonitis, and diabetes mellitus in patients on PD. Correlations between sICAM-1 levels in drained dialysate and body mass index, duration of PD, and age in patients on PD. sICAM-1: soluble intercellular adhesion molecule-1; PD: peritoneal dialysis; NS: not significant.

Levels of sICAM-1 in drained dialysate were not different between men and women (Figure 4). Levels of sICAM-1 were also not different between patients on PD who had episodes of acute inflammatory peritonitis and those who did not (Figure 4). Levels of sICAM-1 in drained dialysate in patients on PD who had diabetes mellitus were significantly higher than those in patients on PD who did not have diabetes mellitus (Figure 4). Levels of sICAM-1 in drained dialysate were significantly negatively correlated with body mass index (*n* = 94, *r* = −.22, *p* < .05) (Figure 4) and positively correlated with the duration of PD (*n* = 94, *r* = .28, *p* < .01) (Figure 4). However, sICAM-1 levels were not correlated with age in patients on PD (Figure 4).

### Correlations between sICAM-1 levels in drained dialysate and serum levels of albumin, C-reactive protein, and blood pressure

**Figure 5.**
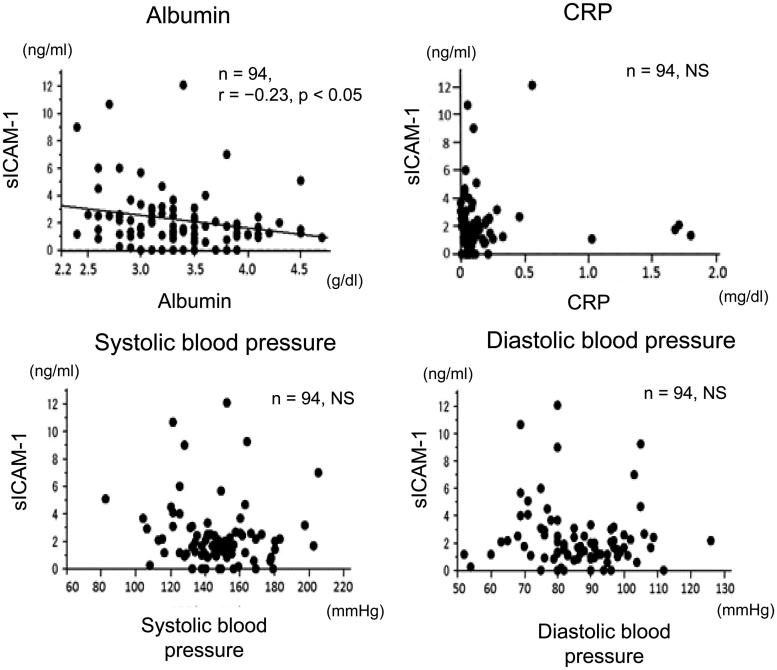
Correlations between sICAM-1 levels in drained dialysate and serum levels of albumin, CRP, and blood pressure in patients on PD. Correlations between sICAM-1 levels in drained dialysate and serum levels of albumin and CRP, and systolic and diastolic blood pressure in patients on PD. sICAM-1: soluble intercellular adhesion molecule-1; PD: peritoneal dialysis; CRP: C-reactive protein; NS: not significant.

Levels of sICAM-1 l in drained dialysate were significantly negatively correlated with serum albumin levels (Figure 5) (*n* = 94, *r* = −.23, *p* < .05). However, sICAM-1 levels were not correlated with serum C-reactive protein (CRP) (Figure 5), and systolic and diastolic blood pressure (Figure 5).

## Discussion

Our study showed that ICAM-1 expression levels were increased in the peritoneum of rats with peritoneal injury. Additionally, sICAM-1 levels in drained dialysate were associated with the solute transport rate, as determined by the PET, and MMP-2 levels in drained dialysate in patients on PD. These results suggest that sICAM-1 levels in drained dialysate reflect peritoneal injury and could be a novel biomarker of peritoneal injury in patients on PD. To the best of our knowledge, this is the first study to demonstrate a significant correlation between sICAM-1 levels in drained dialysate and peritoneal injury in patients on PD.

ICAM-1 is an adhesion molecule, which is expressed in various cells.^10^ ICAM-1 plays a pivotal role in inflammation.^11^ ICAM-1 also facilitates leukocyte adhesion and migration to inflammatory sites by binding lymphocyte function-associated antigen (LFA-1) to leukocytes.^11^ The circulating form of ICAM-1, sICAM-1, is produced by the following two mechanisms: (1) generation from ICAM-1 by shedding from the cell membrane by proteolytic cleavage and (2) translation from messenger RNA transcripts specific for sICAM-1.^12^ ICAM-1 and sICAM-1 are increased in inflammation by pro-inflammatory cytokines.^10^^,^^12^ Because sICAM-1 has been found in various body fluids, it has been reported to be a potentially useful biomarker for various diseases.^12^ In our study, we found that ICAM-1 expression levels were increased in the peritoneum of rats with peritoneal injury, and sICAM-1 levels in drained dialysate were associated with peritoneal injury in patients on PD. These results suggest that sICAM-1 could be a potential useful biomarker for peritoneal injury in patients on PD.

A previous study reported that serum sICAM-1 and CRP levels were correlated in cardiovascular disease because both of them reflected chronic inflammation.^17^ In our study, no correlation was observed between sICAM-1 levels in drained dialysate and serum CRP levels in patients on PD. Because the molecular weight of sICAM-1 is high (240–500 kD),^18^ sICAM-1 does not to pass freely from serum to the peritoneal cavity. Therefore, sICAM-1 in drained dialysate might be produced locally in the peritoneum by chronic inflammation. The high level of ICAM-1 in the peritoneum of rats with peritoneal injury supports this possibility. However, further studies are required to investigate the mechanism of producing sICAM-1 in drained dialysate in patients on PD. Previous studies have reported that serum sICAM-1 levels in patients with diabetes mellitus are significantly higher than those of patients without diabetes mellitus.^19^ Another study reported that serum sICAM-1 levels were negatively correlated with serum albumin levels.^17^ This previous study suggested that there is a positive association between malnutrition and chronic inflammation, which increases serum sICAM-1 levels. In the current study, sICAM-1 levels in drained dialysate of patients on PD with diabetes mellitus were higher than those of patients on PD without diabetes mellitus. Additionally, sICAM-1 levels in drained dialysate were negatively associated with serum albumin levels and body mass index. These results are consistent with those of previous studies.^17^^,^^19^ Our results suggest the peritoneal injury by chronic inflammation progressed with the condition of diabetes mellitus and malnutrition.

In our study, sICAM-1 levels in drained dialysate were correlated with the duration of PD. This finding suggested that peritoneal injury might have progressed in accordance with the duration of PD. Repeated exposure of PD solution and indwelling PD catheters may contribute to chronic inflammation of the peritoneum leading to peritoneal injury in patients on PD. Further studies are required to investigate the factors and mechanisms of regulation of sICAM-1 in drained dialysate in patients on PD.

In addition to the role of ICAM-1 as an adhesion molecule, it also functions as a signal transduction molecule for developing chronic inflammation by binding LFA-1 to leukocytes.^20^ However, the roles of sICAM-1 in inflammation are not well known. sICAM-1 is capable of inhibiting leukocyte migration and signal transduction via ICAM-1 and LFA-1 interaction by binding LFA-1 to leukocytes.^21^ Additionally, sICAM-1 may promote angiogenesis, which is closely associated with inflammation.^22^ The detailed roles of ICAM-1 and sICAM-1 for developing peritoneal injury merit further investigation. In conclusion, ICAM-1 expression is increased in the peritoneum of a peritoneal injury model in the rat, and sICAM-1 levels in drained dialysate are associated with peritoneal injury in patients on PD. These results suggest that sICAM-1 could be a novel biomarker of peritoneal injury in patients on PD.
